# Effect of ankle-foot orthosis on functional mobility and dynamic balance of patients after stroke: Study protocol for a randomized controlled clinical trial

**DOI:** 10.1097/MD.0000000000017317

**Published:** 2019-09-27

**Authors:** Gabriela Vieira de Paula, Taís Regina da Silva, Juli Thomaz de Souza, Gustavo José Luvizutto, Silméia Garcia Zanati Bazan, Gabriel Pinheiro Modolo, Fernanda Cristina Winckler, Letícia Cláudia de Oliveira Antunes, Luís Cuadrado Martin, Rafael Dalle Molle da Costa, Rodrigo Bazan

**Affiliations:** a Rehabilitation Department; b Department of Internal Medicine; c Department of Applied Physical Therapy. Federal University of Triângulo Mineiro (UFTM), Uberaba, MG-BRA; d Department of Neurology, Psychology and Psychiatry. Sao Paulo State University (UNESP). Botucatu Medical School, Botucatu, SP-BRA, Brazil.

**Keywords:** gait, orthoses, postural balance, rehabilitation, stroke

## Abstract

**Introduction:**

Stroke is the principal cause of impairment in the motor function and gait of adults. One of the resources used in rehabilitation to optimize gait is a prescription of ankle-foot orthosis (AFO), and the most prescribed AFOs are Fixed AFO and Articulated AFO; however, it is not known which of these options is more effective for these individuals. The aim of this study is to evaluate the impact of different types of ankle-foot orthosis functional mobility and dynamic balance in stroke patients.

**Methods:**

Prospective randomized controlled clinical trial with 2 parallel groups will be conducted, and the aim is to recruit 50 patients with stroke diagnosis within 1 year, who indicated that they use both types of orthotics, who had a previous Rankin score less than or equal to 3, and who obtained AFO orthosis through the Hospital Clinics at the Botucatu Medical School (HCBMS) São Paulo, Brazil. After a specific evaluation by a physiotherapist, the patient will receive 1 of the AFO types via randomization. After 30 days, the patient will be reevaluated. The primary outcomes will be balance and mobility, which will be evaluated by the Time Up Go Test (TUG) and Tinetti's Scale of Mobility and Balance (TSMB). The secondary outcomes will be quality of life and the levels of anxiety and depression, which will be evaluated with the European (5D) Quality of Life Scale (Euroqol) and Hospital Anxiety and Depression Scale (HADS). Group allocation will be not concealed because the blinding of participants and of therapists that provide intervention is not possible, and all analyses will be based on an intention-to-treat principle. This study was approved by the Human Research Ethics Committee of the São Paulo State University UNESP, number 2.367.953. The results will be published in relevant journal.

**Discussion:**

The results of this study will contribute to clinical practice by identifying the type of AFO orthosis that is more suitable for this condition, helping to standardize prescription of these orthoses by professionals, and guiding future research studies on this subject, which is still incompletely defined in the literature.

**Trial registration:**

RBR-6SF2VV (March 5, 2018).

## Abstract 2

Supplemental Digital Content is available in the text

## 1 Introduction

Stroke is the second most common cause of death in the world and generates high levels of functional incapacity in the chronic phase.^[1]^

Stroke provokes innumerable alterations in motor function, and hemiplegia is the most frequent manifestation after the event.^[2]^ Several studies have revealed that muscular strength and tonus present modifications after stroke according to the location and severity of the injury to the cerebral tissue.^[3]^ Besides these alterations, impairments in the visual, vestibular, and sensorial systems as well as coordination may also be present in these individuals.^[4]^

Thus, the modifications caused after stroke in hemiplegic individuals are also reflected in gait execution, which presents a marked reduction in velocity, postural asymmetry, and disturbances in balance and in postural control.^[5]^

The incapacities and diverse modifications resulting from stroke require that affected individuals make substantial adaptations in order to deal with the situation.^[6,7]^ The presence of depression in this population is very frequent, and in many cases, depression can impede adherence to treatment and progress for these individuals.^[8]^

Gait deficits present a negative impact on post-stroke functional capacity by impairing the perception of these individuals in relation to their functionality and social participation as well as potentially leading to prejudice about their quality of life in this population.^[9,10]^ A study of individuals after stroke demonstrated that functionality and mobility, when they are impaired, negatively influenced life quality, and the individuals with diminished velocity and quality of gait presented worse quality-of-life scores.^[11]^

One of the assistive technology resources employed by physiotherapy to optimize the gait after stroke is the ankle-foot orthosis (AFO). It is an external device utilized on a lower limb to stabilize the joints and provide a more adequate gait. It may be prescribed in any period of rehabilitation and can be substituted or modified according to necessity or evolution.^[12]^

The first references to the use of thermoplastic materials in the construction of orthoses for lower limbs were published at the end of the 1960s by the pioneers Yates and Lehneis.^[13,14]^ Lehneis et al studied the biomechanics of thermoplastic AFO and recognized the enormous potential of using these materials in orthoses for lower limbs.^[15]^ Over the years, the models and application of AFOs have continued to evolve, including the ones that are currently in use.^[16]^

One of the principal post-stroke problems during gait is equinus deformity, which is generally associated with spasticity and shortening of the sural triceps and results in dorsiflexion reduction.^[17]^ Spasticity of the sural triceps can also lead to another abnormal manifestation during gait, specifically hyperextension of the knee in the support phase. This manifestation can also be minimized via the use of AFO orthosis, which diminishes the hyperactivity of the sural triceps upon neutralizing the foot and cooperates in the correction of equinus deformity and knee hyperextension.^[18]^ Furthermore, it presents improvement in balance and diminishes fall risks in patients who present signs of hemiplegia.^[19]^

The 2 types of AFO orthoses most often prescribed after stroke are Fixed AFO and Articulated AFO. The Fixed AFO is indicated to eliminate excessive plantar flexion and minimize hyperextension of the knee.^[18]^ It is also utilized to prevent joint deformities in non-ambulatory patients.^[12]^

The Articulated AFO provides aid in dorsiflexion, blocking the ankle in an adequate position; by being articulated, it permits ankle movements, unlike Fixed AFO, which neutralize this joint, and it also corrects a hyperextended knee.^[20]^ Nevertheless, there are few studies that have evaluated the use of different types of AFO orthoses in stroke patients. The prescription of the AFO-type orthosis for these patients ends up occurring frequently by professionals due to the small number of studies available on this topic.

Due to their high incapacitating potential, strokes have a great impact on public health^[21]^; therefore, more studies are necessary to verify the most efficacious treatments for this population. In relation to the type of AFO for this condition, the literature offers little information. Specifically, more research studies are necessary to determine which orthosis presents greater efficacy in this pathology. Our main hypothesis is that the distinct AFO types differ in the recovery of motor performance in patients after stroke. We hypothesized that Fixed AFO improves hemiplegic gait, but by not limiting the normal movements of the ankle, Articulated AFO would be more beneficial in the rehabilitation of these patients.

## 2 Objective

We aim to evaluate the impact of the type of AFO orthosis on motor function and gait in individuals after a stroke. We will also investigate the relationship between the prescribed AFO orthosis type and life quality, anxiety, and depression.

## 3 Methods/design

### 3.1 Trial design

**Figure 1:**
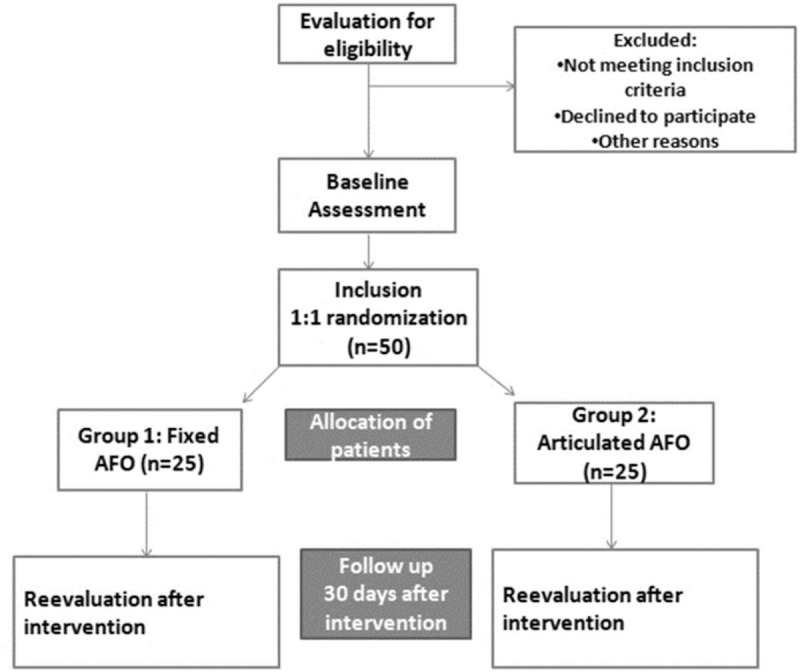
Study flowchart.

This single-center, open, randomized, controlled clinical trial with parallel groups of 50 patients after stroke will be conducted according to the flow diagram and will last for 12 months, scheduled to start in May 2019 (Fig. 1).

### 3.2 Location and setting

All assessments will be conducted at the Botucatu Medical School within the Department of Neurology and Center of Rehabilitation, Botucatu, Brazil.

### 3.3 Participants

#### 3.3.1 Eligibility criteria

The patients recruited for the study will be recruited from the Rehabilitation Sector of the Hospital of Clinics at the UNESP Botucatu Medical School, and the inclusion criteria will be verified.

The patients who have an indication for the use of both AFO types are those with hemiplegia or hemiparesis who present diminished walking ability, postural control alterations, diminished joint mobility (principally in dorsiflexion), balance disorders, muscular tonus alterations with varo-equinus deformities, presence of knee hyperextension in the support phase and impairment of proprioception in some cases.^[22]^

The inclusion criteria for the study are as follows:

- Individuals of either sex who can utilize both types of orthoses in treatment;
- Less than 1 year from stroke;
- A previous Rankin score of less than or equal to 3;
- An indication of AFO orthosis after evaluation of ambulatory rehabilitation at the Hospital Clinics at the UNESP Botucatu Medical School.

#### 3.3.2 Exclusion criteria

Individuals with amputation of a lower limb and/or being a carrier of a progressive neuromuscular disease.

#### 3.3.3 Discontinuing criteria

Any patients who do not use the orthosis and/or have dermatitis in the hemiplegic leg.

### 3.4 Procedures (Template)

Post-stroke patients will be evaluated by a physiotherapist and a physician with experience in the evaluation and prescription of AFOs. After evaluation by the team, the patients who have an indication for the use of AFO orthosis and fit the eligibility criteria of the study will be referred for specific evaluation. All the participants will sign terms of free and informed consent for participation in the study.

The individuals who fit the criteria for inclusion in this study will be randomized to receive 1 of the 2 types of orthoses: Fixed AFO or Articulated AFO.

The first evaluation will occur before the patient begins the use of AFO orthosis, and then the patient will be reevaluated 30 days after the intervention (AFO use).

All the scales, questionnaires, and tests that will be applied were already translated, adapted and validated for the Portuguese language used in Brazil. Individuals will be assessed using the Time Up Go Test (TUG), Tinetti's Scale of Mobility and Balance (TSMB), European (5D) Quality of Life Scale (Euroqol), Hospital Anxiety and Depression Scale (HADS), modified Rankin scale (mRs), National Institute of Health Stroke Scale (NIHSS), Barthel Index (BI), and the Modified Scale of Ashworth (mAS).

### 3.5 Randomization and blinding

The process of randomization will be performed using Microsoft Excel for Windows by a researcher who was not involved in the recruitment of participants. The participants will be randomized into 2 groups at a proportion of 1:1 with 25 patients allocated into each group. After evaluation, the participants who are eligible for the study will be referred to a physiotherapist responsible for their specific evaluation.

Because of the nature of the interventions, it will be impossible to blind the therapists and patients involved in the study.

### 3.6 Intervention

After specific evaluation by a physiotherapist, the patient will receive 1 of the 2 types of AFOs according to the randomization either Fixed or Articulated.

Group 1: Fixed AFO

The Fixed AFO orthosis neutralizes the ankle by blocking movements, eliminating excessive plantar flexion, and minimizing knee hyperextension in the medial support phase.^[18]^

Group 2: Articulated AFO

Articulated AFO orthosis provides aid in dorsiflexion, leaves the ankle in an adequate position, and allows its movement by virtue of being articulated.^[20]^

The patients will be instructed about the correct placement and use of the orthosis; furthermore, a member of the team will make weekly telephone contact to verify adherence to the treatment. After 30 days, the patient will be reevaluated by applying the scales and tests that were previously mentioned.

All patients underwent physiotherapy during the study period.

### 3.7 Primary outcome measures

The primary outcomes will be balance and mobility, which will be evaluated by the TUG and by TSMB. These outcomes will be assessed after the intervention.

### 3.8 Secondary outcome measures

The secondary outcomes will be the quality of life and the levels of anxiety and depression after the intervention, which will be evaluated by the Euroqol and HADS, consecutively. Additional secondary outcomes include the incapacity level, the degree of neurological deficit severity, the functional capacity for basic daily life activities and spasticity of the lower limb affected after the stroke, which were evaluated using the mRs, NIHSS, BI, and mAS.

### 3.9 Sample size calculation

The sample will be composed of 50 volunteers randomized into 2 groups to receive 1 of 2 types of AFO. This number of participants are capable to detecting a 30% difference in improvement of the primary outcome (TUG and TSMB) with an alpha error of 0.05 and beta error of 0.2.

### 3.10 Baseline assessments

#### 3.10.1 Sociodemographic evaluation

Sociodemographic data will be collected from the participants, as well as data on currently used medications, previous diseases, and previous physiotherapy treatments.

### 3.11 Neurological and functional evaluation

The participants will be classified according to the incapacity that the stroke caused by mRs. The scoring on this scale varies from 0 to 6, with 0 indicating lack of incapacity and 6 indicating fatality.^[23]^

The NIHSS is widely utilized, valid, and reliable. It enables quantification of the severity of neurological deficits after stroke and evaluates 11 items: consciousness level, ocular deviation, facial paresis, language, speech, negligence, motor function, limb sensitivity and ataxia. Its scoring varies from 0 (no evidence of neurological deficits for the tested sphere on the scale) to 42 (patient in a coma and unresponsive).^[24]^

The Euroqol is a validated scale that includes 5 questions addressing mobility, personal care, usual activities, pain/discomfort and anxiety/depression. The patients will respond according to their perception of health and then will indicate their perception on a ruler from 0 to 100 with 0 and 100 signifying the worst and best health status, respectively.^[25]^

The HADS scale briefly evaluates the levels of anxiety and depression in individuals with physical pathology and in ambulatory treatment; it is composed of 14 questions, including 7 on depression and 7 on anxiety.^[26]^

The TSMB assesses the performance of balance and the presence of alterations in the gait. It is composed of 16 items observed by the evaluator, including 9 for body balance and 7 for gait. It classifies aspects of gait, such as velocity, stepping distance, symmetry, 360° gyrations, balance, and changes with the subject's eyes closed. The score on each item varies from 0 to 1 or from 0 to 2 in which the lower the score, the poorer is the physical ability. The maximum score is 16 points for balance and 12 for gait with a total of 28 points. Scores below 19 points represent a high risk of falling, while those between 19 and 24 points represent a moderate risk of falls.^[27]^

The TUG test is utilized for evaluation of functional mobility involving velocity, potency, and dynamic balance. It is carried out in the following manner: patients will be seated in a chair, and then, utilizing a stopwatch, a measurement will be made of the time they require to stand up, walk a distance of 3 m, turn around, return, and sit again. The individual will be allowed to use auxiliary gait devices if necessary. The shorter the time to execute the test, the better is the functional performance.^[28]^

The BI measures the degree of assistance the individual requires in 10 activities: feeding oneself, bathing oneself, personal care, dressing oneself, controlling their urinary sphincter, controlling their intestinal sphincter, using the bathroom, executing bed-chair transferals, walking, and climbing a flight of stairs. The scoring varies from 0 to 100, and higher scores indicate a greater degree of functional independence.^[29]^

Spasticity of the affected lower limb will be evaluated by mAS, which is a scale that grades spasticity according to the movement of a muscle group and its resistance to joint movement; its scoring varies from 0 to 4, and the higher the score, the higher is the spasticity severity.^[30]^

### 3.12 Confounding variables

This analysis will be adjusted for potential confounders, such as age, sex, mRs at discharge, NIHSS scores, and type of stroke.

### 3.13 Statistical analysis

The analyses will follow the principle of Intention to Treat. The Kolmogorov-Smirnov test will be employed to verify the normality of the distribution. The non-categorical data of a parametric distribution will be analyzed by two-way ANOVA for repeated measures, and multiple comparisons will be analyzed with the Tukey test. The categorical data at the baseline moment will be compared using the Chi-Squared test. The nonparametric distribution variables will be categorized as improvement or worsening and compared between groups with the Chi-Squared test. The non-categorical data will be expressed as means and standard deviations, and the categorical data will be expressed as percentages. Statistical significance will be established as *P* < .05.

### 3.14 Patient and public involvement

Patients and/or public were not involved in the design of this study.

## 4 Discussion

Stroke is the principal incapacitating disease in adults,^[1]^ and the importance of optimizing strategies to rehabilitate patients affected by this disease is evident. Mobility improvement is the primary objective for most patients and therapists since it is essential for the independence of these individuals.^[31]^

Several studies have shown that AFO use produced improvement in the cadence and velocity of the gait by minimizing knee hyperextension along with improvements in symmetry and stability in these patients.^[32]^ Although there are some studies that report efficacy of AFO in these hemiplegic patients, research on its effects in relation to mobility, and to balance is scarce^[33]^; furthermore, there is no consensus on which AFO model is more efficacious for treating this pathology.

The results of this study will contribute to clinical practice by identifying the type of AFO orthosis that is more suitable for this condition, helping to standardize prescription of these orthoses by professionals, and guiding future research studies on this subject, which is still incompletely defined in the literature.

Our goal is to accomplish a randomized clinical trial of high quality that utilizes validated evaluation measures not only for balance and mobility in our primary outcomes but also for the levels of anxiety and depression in our secondary outcomes. If 1 of the AFO types is proven to be superior to the other in some of these aspects, this evidence would permit professionals to recommend the ideal AFO for patients with stroke sequelae, which will minimize their incapacity and optimize their rehabilitation. For our results to generate an impact on the clinical practice of therapists and on patient care, they should be publicized at congresses and in academic journals pertaining to this research area.

## 5 Trial status

At the time of submission, recruitment was ongoing. Recruitment started on March 15, 2018 and is expected to be completed on November 30, 2019. It was registered by Brazilian Registry of Clinical Trials ReBEC (TRIAL: RBR-6SF2VV) on March 08, 2018 and is being financed by National Council for Scientific and Technological Development (CNPq) through process number 423924/2016-8.

(Appendices:).

## Acknowledgments

We are grateful to FMB-UNESP (Botucatu Medical School from São Paulo State University and HC-FMB (Botucatu Medical School Clinical Hospital) for allowing the use of their facilities.

## Author contributions

**Conceptualization:** Rodrigo Bazan.

**Data curation:** Gabriela Vieira de Paula, Taís Regina da Silva, Letícia Cláudia de Oliveira Antunes.

**Investigation:** Gabriela Vieira de Paula, Taís Regina da Silva.

**Methodology:** Juli Thomaz de Souza, Rodrigo Bazan.

**Supervision:** Luís Cuadrado Martin, Rafael Dalle Molle da Costa, Rodrigo Bazan.

**Writing – original draft:** Gabriela Vieira de Paula.

**Writing – review & editing:** Taís Regina da Silva, Juli Thomaz de Souza, Gustavo José Luvizutto, Silmeia Zanati Bazan, Gabriel Pinheiro Modolo, Fernanda Cristina Winckler, Luís Cuadrado Martin, Rafael Dalle Molle da Costa, Rodrigo Bazan.

Juli Thomaz de Souza orcid: 0000-0003-2227-7505.

## Supplementary Material

Supplemental Digital Content
