# Supplementary material for: Effect of ankle-foot orthosis on functional mobility and dynamic balance of patients after stroke: Study protocol for a randomized controlled clinical trial
Source: Medicine (Baltimore). 2019 Sep 27;98(39):e17317. doi: 10.1097/MD.0000000000017317 (PMC6775434; doi:10.1097/MD.0000000000017317)
Supplement: Supplemental Digital Content [file medi-98-e17317-s001.doc]

**APÊNDICE 1 - TERMO DE CONSENTIMENTO LIVRE E ESCLARECIDO (TCLE)**

CONVIDO, o Senhor (a) para participar do Projeto de Pesquisa intitulado **“EFEITO DA ÓRTESE TORNOZELO-PÉ NA MOBILIDADE FUNCIONAL E EQUILÍBRIO DINÂMICO DE PACIENTES APÓS ACIDENTE VASCULAR CEREBRAL: ENSAIO CLÍNICO CONTROLADO RANDOMIZADO”**, que será desenvolvido por mim **Gabriela Vieira de Paula**, aluna de mestrado, com orientação do **Prof.Dr Rodrigo Bazan** professor da Faculdade de Medicina de Botucatu – UNESP; e será realizado no Setor de Reabilitação do Hospital das Clínicas da Faculdade de Medicina de Botucatu.

Estou avaliando o desempenho motor (velocidade e equilíbrio) do membro inferior afetado, durante a marcha com a órtese de pacientes após a ocorrência do Acidente Vascular Cerebral. Para que eu possa ter um resultado nesse momento preciso realizar uma avaliação com os dados pessoais, histórico da doença, medicamentos em uso, hábitos de vida, aplicação do teste equilíbrio de Tinetti (EMET), que é um questionário que avalia o equilíbrio, o teste time up go (TUG); onde o paciente estará sentado em uma cadeira, será cronometrado o tempo em que ele levantar-se, andar uma distância de três metros, virar-se, caminhará e voltará novamente a sentar-se novamente, será aplicada também a escala *Hospital* Anxiety Depression Scale (HADS) que detecta a presença de depressão, uma escala de qualidade de vida com 5 perguntas chamada Euroqol, as escalas Rankim modificada (ERm) e NIH Stroke Scale que irão avaliar a gravidade e o grau de comprometimento após acidente vascular cerebral. Será também a graduação da espasticidade do membro inferior que é para graduar a rigidez após o AVC.

O Senhor (a) responderá um questionário que levará uns 50 minutos de duração, incluindo as avaliações acima citadas e as escalas. Todo procedimento será realizado nesse único dia.

Seu benefício em participar será a conscientização de seu desempenho motor para realização das atividades de vida diária.

Fique ciente de que sua participação neste estudo é voluntária e que mesmo após ter dado seu consentimento para participar da pesquisa, você poderá retira-lo a qualquer momento, sem qualquer prejuízo na continuidade do seu tratamento.

Este Termo de Consentimento Livre e Esclarecido será elaborado em 2 vias de igual teor, o qual 01 via será entregue ao Senhor (a) devidamente rubricada, e a outra via será arquivada e mantida pelos pesquisadores por um período de 5 anos após o término da pesquisa.

Qualquer dúvida adicional você poderá entrar em contato com o Comitê de Ética em Pesquisa através dos telefones (14) 3880-1608 ou 3880-1609 que funciona de 2ª a 6ª feira das 8.00 às 11.30 e das 14.00 às 17horas, na Chácara Butignolli s/nº em Rubião Júnior – Botucatu - São Paulo. Os dados de localização dos pesquisadores estão abaixo descrito:

Após terem sido sanadas todas minhas dúvidas a respeito deste estudo, CONCORDO EM PARTICIPAR de forma voluntária, estando ciente que todos os meus dados estarão resguardados através do sigilo que os pesquisadores se comprometeram. Estou ciente que os resultados desse estudo poderão ser publicados em revistas científicas, sem no entanto, que minha identidade seja revelada.

Botucatu,_____/___/_____ _____________________ _________________________

Pesquisador Participante da Pesquisa

Nome: Gabriela Vieira de Paula

Nome: Rodrigo Bazan

Telefone:

Email:

Telefone: (18) 996412568

Email: gvieiradepaula@gmail.com
